# Hyperspectral detection of fresh corn peeling damage using germinating sparse classification method

**DOI:** 10.3389/fpls.2022.1039110

**Published:** 2022-11-29

**Authors:** Zhenye Li, Jun Fu, Zhi Chen, Qiankun Fu, Xiwen Luo

**Affiliations:** ^1^ Key Laboratory of Bionic Engineering, Ministry of Education, Jilin University, Changchun, China; ^2^ Key Laboratory of Efficient Sowing and Harvesting Equipment, Ministry of Agriculture and Rural Affairs, Jilin University, Changchun, China; ^3^ Chinese Academy of Agricultural Mechanization Sciences, Beijing, China; ^4^ College of Engineering, South China Agricultural University, Guangzhou, China

**Keywords:** fresh corn, hyperspectral image, dictionary learning, sparse representation, damage detection

## Abstract

Peeling damage reduces the quality of fresh corn ear and affects the purchasing decisions of consumers. Hyperspectral imaging technique has great potential to be used for detection of peeling-damaged fresh corn. However, conventional non-machine-learning methods are limited by unsatisfactory detection accuracy, and machine-learning methods rely heavily on training samples. To address this problem, the germinating sparse classification (GSC) method is proposed to detect the peeling-damaged fresh corn. The germinating strategy is developed to refine training samples, and to dynamically adjust the number of atoms to improve the performance of dictionary, furthermore, the threshold sparse recovery algorithm is proposed to realize pixel level classification. The results demonstrated that the GSC method had the best classification effect with the overall classification accuracy of the training set was 98.33%, and that of the test set was 95.00%. The GSC method also had the highest average pixel prediction accuracy of 84.51% for the entire HSI regions and 91.94% for the damaged regions. This work represents a new method for mechanical damage detection of fresh corn using hyperspectral image (HSI).

## 1 Introduction

Fresh corn is favored by consumers for its excellent nutritional value and edible quality (Saka et al., 2018). Mechanical peeling is the main link in the processing of fresh corn, and the collision, extrusion, and friction between the ear and the high-speed rotating peeling roller can easily cause the mechanical damage of varying degrees to the crisp and tender kernels (Zhao et al., 2011). At present, most fresh corn processing plants still use the manual method to select and grade damaged ears. However, manual grading is a tedious and inefficient work, and it is more difficult to observe with naked eyes in the case of slight abrasion of seed coats (Wang, 2010). Therefore, it is urgent to find a fast automatic detection technology to detect the damage of fresh corn ear.

For detection of damaged corn ears, researchers identified them based on RGB images (Fu et al., 2020), and constructed a classification model using the color characteristics and texture characteristics (Zhang F et al., 2015). The detection of damaged corn based on traditional RGB images mainly utilizes the color difference and spatial feature difference of the target, and the classification results are seriously affected by the image acquisition environment (Gao and Liu, 2016). HSI data is a type of three-dimensional cube containing spatial pixel information (two-dimensional) and spectral information (one-dimensional) of an object (Torres et al., 2019). The wavelength of each pixel covers the entire spectral range, and meanwhile, all the data in each wavelength can form an image (Yin et al., 2017). Hence, HSI can extract more feature information for more complex spectral analysis and image processing, which cannot be achieved by the traditional machine vision. With the development of optical sensors and imaging technology, more and more studies have reported the application of HSI in the quality assessment and safety detection of various agricultural products (Kandpal et al., 2015; Su et al., 2021; Nazir et al., 2022). Among them, in the aspect of damage detection, HSI has been successfully used for the detection of crack in fresh jujube (Yu et al., 2014), scratch, scar, and spot in peach (Zhang B et al., 2015), insect damage in soybeans (Huang et al., 2013), wheat kernels damaged by Fusarium head blight (Lv et al., 2022), and freeze-damage of corn seed (Zhang et al., 2019). As far as we know, there is no report on the damage detection of fresh corn ear based on HSI. Therefore, an investigation into the potential of detecting the peeling-damaged regions of fresh corn with the spectral range of 400 nm–1000 nm deserves special attention.

At present, methods of partial least squares discriminant analysis (PLS-DA) (Ambrose et al., 2016), support vector machine (SVM) (Wakholi et al., 2018), K-singular value decomposition (K-SVD) (Fang et al., 2015), random forest (RF) (Che et al., 2018), and artificial neural networks (ANNs) (Zhang et al., 2021) are often used for HSI classification and have achieved relatively good classification results. However, a study of existing classification methods found that there are still some deficiencies in the current researches, which can be summarized as follows: (1) Non-machine-learning methods are limited by unsatisfactory detection accuracy. (2) Machine-learning methods, especially deep learning methods, rely heavily on training samples. However, there usually exist some interference samples while building training datasets, which significantly influence the accuracy of machine learning. (3) The background contents are not fixed in different detecting situations, containing various features, and are difficult to be accurately classified. Moreover, it is more difficult to classify the sound and damaged regions of fresh corn than other agricultural products due to the large number of kernels on the ear, the vertical and horizontal gaps between kernels, and the interspersed corn whisker. Therefore, it is necessary to develop a suitable algorithm for identifying the damage regions of fresh corn.

Aiming at the shortcomings of current methods, the germinating sparse classification (GSC) method was proposed to detect the sound and peeling-damaged regions of fresh corn. The innovations of this method could be summarized as follows: (1) The ‘seed germinating’ strategy is developed to refine training samples, which reduces the influence of unqualified training samples on dictionary learning. (2) For dictionary learning process, the atoms that are incompetent to accurately represent any training samples are removed during iterations, and the number of atoms can be dynamically adjusted. The strategy improves the performance of dictionary and reduces computational complexity. (3) For sparse classification process, the threshold algorithm with respect to the energy of sparse representation residual is proposed to determine the pixels of background. In addition, three commonly used classification methods K-SVD, SVM, and back propagation (BP) neural network were used to classify the sound, peeling-damaged, and background pixels in fresh corn hyperspectral images (HSIs). The classification results of the three methods were compared with that of the proposed GSC method to verify the superiority of the GSC method in the detection of fresh corn peeling damage.

## 2 Experimental materials and data

In this section, the fresh corn materials and equipment used in this study are described. Next, the hyperspectral data acquisition and preprocessing are introduced.

### 2.1 Fresh corn materials

The fresh corn variety used in this study was Jinxiangnuo, which was widely cultivated in Northeast China. One hundred and eighty fresh corn ears were hand-selected from the fresh corn production line at Fumin Food Processing Plant in Songyuan city, Jilin Province, China. The fresh corn ears were inspected by five trained workers and divided into sound and peeling-damaged. The 180 fresh corn ears were composed of 90 sound ears and 90 peeling-damaged ears. The hyperspectral data of randomly selected 60 sound ears and 60 peeling-damaged ears formed the training set, and the hyperspectral data of the remaining ears constituted the test set. The selected fresh corns were sealed in airtight bags, then stored at the optimum condition of 4°C to retain moisture. All fresh corn HSIs were taken within two hours after peeling.

### 2.2 Hyperspectral image acquisition

**Figure 1:**
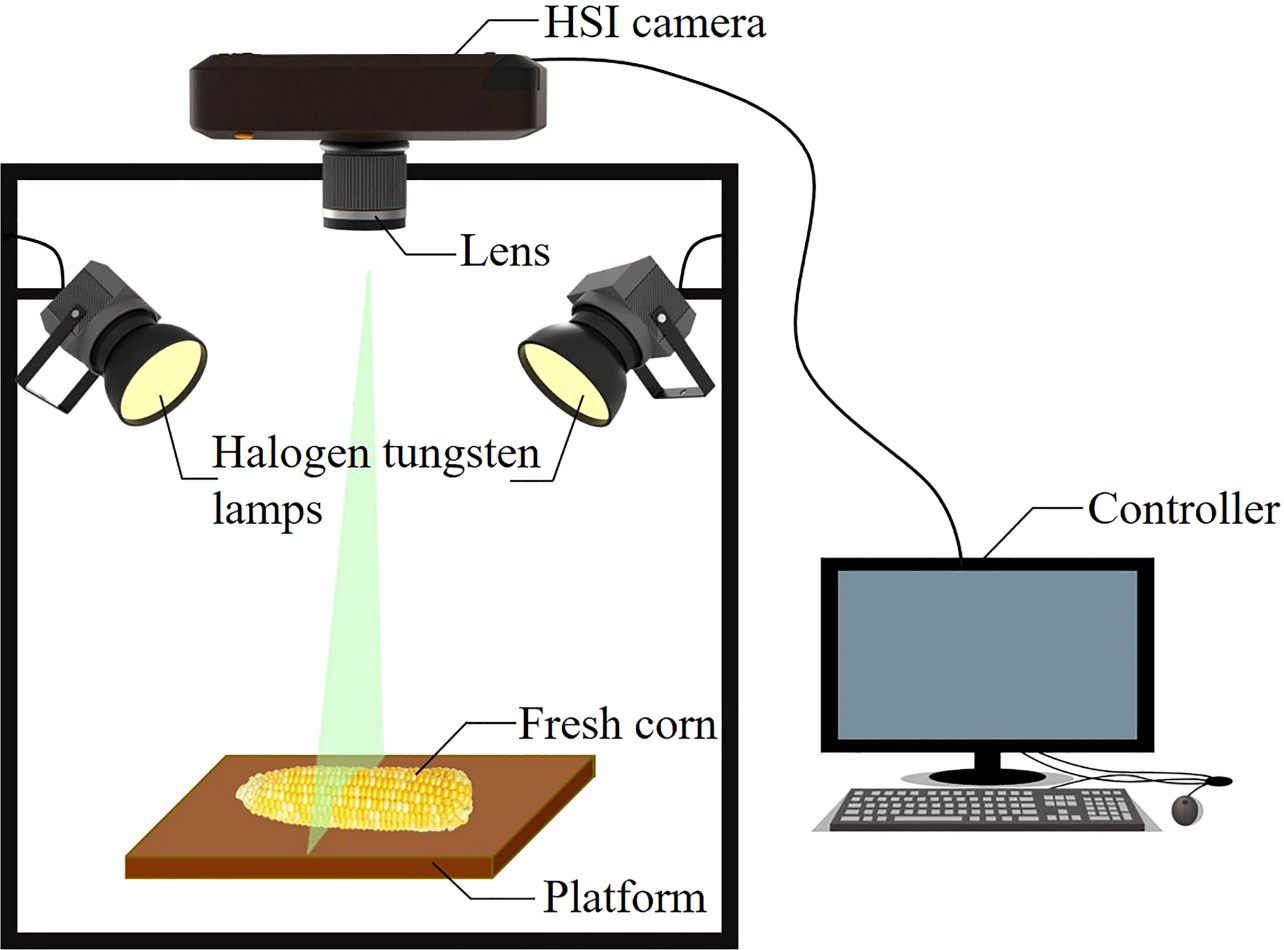
Schematic diagram of the hyperspectral imaging system for acquiring reflectance images of fresh corns.

Hyperspectral data of fresh corn ears were collected by using a hand-held visible-NIR hyperspectral imaging system (Specim IQ, Specim Ltd., Finland). The system integrated a hyperspectral camera, scanning platform, image acquisition card, data acquisition software, and data processing software. As presented in **Figure 1**, the hyperspectral imaging system kept the internal environment consistent during all the acquisition processes to reduce the interference from the outside. The imaging spectrograph covered a spectral range of 400 nm–1000 nm. The resulting hyperspectral data cube had dimensions of 512×512 pixels and 204 wavebands. The lighting system consisted of two 150 W halogen tungsten lamps (QVF133, Philips Lighting (Shanghai) Co., LTD., Shanghai, China) which were fixed on both sides of the test platform at an angle of 45°. The hyperspectral imaging system operated at an exposure time of 22 ms during data acquisition. The distance between the lens and the fresh corns was 38 cm.

### 2.3 Hyperspectral data preprocessing

HSIs need to be calibrated by using white and dark reference images. The raw HSI was calibrated using equation (1):


(1) $$R_{c}=\frac{R_{o}-R_{b}}{R_{w}-R_{b}}$$

where, *R_c_* is the calibrated HSI; *R_o_* is the raw HSI; *R_b_* is a dark reference image obtained by completely blocking the lens with an opaque cover; *R_w_* is a white reference image of a pure Teflon whiteboard (Spectralon, Labsphere Inc, North Sutton, NH, USA) with 99% reflectivity, obtained under the same environment as the raw HSI.

The RGB images corresponding to fresh corn HSIs were collected, and the region of interest (ROI) of fresh corn were labeled at pixel level by human judgment. The pixels corresponding to the sound regions and damage regions on the corn were denoted as the sound class and peeling-damaged class, respectively. However, it was inevitable to generate some impure training samples because of human mistakes or limited labeling conditions, which would directly affect the discriminant performance of the subsequent models and lead to a reduction of the classification accuracy. In order to improve the purity of training samples, all samples in training sets of the two classes were refined. The detailed method is described in subsection 3.2.2.

Modeling with all-waveband data not only takes a long time to compute and occupies a large amount of memory, but also degrades the performance due to the curse of dimensionality. Therefore, the dimension of hyperspectral data was reduced before using for training in this study. To determine the required spectral wavebands, the dimensionality reduction test including two steps was executed. By given the starting waveband and the ending waveband, first, the average spectra of the refined training samples of the sound class and peeling-damage class were calculated, respectively. Second, the spectral angle was obtained from the average spectra of the two classes of training samples. The spectral angle is utilized to reflect the difference between two classes of spectra. A larger spectral angle indicates greater difference between the two classes of training samples, and implying better performance for classification. Through the results of test presented in **Table 1**, it can be noted that the spectral angle of the two classes of training samples obtained by using the first 70 wavebands (corresponding wavelength range from 400 nm to 607 nm) was the largest, implying that the first 70 bands may have better potential to provide satisfactory classification performance.

**Table 1:** Spectral angles of average spectra of refining samples of two classes in terms of different used bands.

Starting waveband	Ending waveband	Number of used wavebands	Spectral angle
1	204	204	0.103
1	150	150	0.133
1	90	90	0.198
1	70	70	**0.231**
21	90	70	0.164
41	110	70	0.042
1	50	50	0.221

The bold number represents the maximum value of the spectral angles.

The spectra after 70 wavebands (corresponding wavelength range from 608 nm to1000 nm) were mainly infrared and near-infrared, which was not helpful to the damage identification of fresh corn and even interfered with the classification results. Moreover, the complexity of our algorithm was low, and processing 70 wavebands would not significantly increase the running speed. The selected 70 wavebands were universal to both the training sample and the test target. Therefore, the first 70 wavebands were selected and applied to the four classification methods.

## 3 Experimental methods

In recent years, there has been increasing interest in sparse representation of signals. Sparse representation is widely used in the computer vision and pattern recognition in various fields, including image denoising (Sun et al., 2014), image classification (Zheng et al., 2020), face recognition (Liu et al., 2019), disease recognition (Feng and Zhou, 2016), and target tracking (Ma and Xu, 2021), etc. In these applications, the sparse representation method often leads to the most advanced performance. Therefore, this study aimed to develop a classification method based on sparse representation for fresh corn peeling damage. This section briefly reviews the theoretical background of the HSI classification based on sparse representation, and then introduces the detailed processes of the GSC method proposed in this paper.

### 3.1 Related work

In the sparse representation classification (SRC), pixels in the same class are assumed to approximately lie in the same low-dimensional subspace. Suppose there are *C* distinct classes in HSI, and the *c*-th class has *M_c_* training samples. $M=\sum_{c=1}^{C}M_{c}$ is the total number of training samples. A spatial pixel in HSI can be approximately represented as ***x*** =[*x*_1_,*x*_2_,⋯,*x*_*N*_]∈*ℝ* ^*N*×1^ , where *N* is the number of wavebands. The subdictionary ***D***_c_∈*ℝ* ^*N*×*M*_c_^ is constructed by directly extracting the pixels of the *c*-th class in original HSI. All subdictionaries for *C* classes can be obtained, and all these subdictionaries constitute the final dictionary ***D*** =[***d***_1_, ***d***_2_,⋯, ***d***_*c*_,⋯, ***d***_*C*_]∈*ℝ* ^*N*×*M*^ . The SRC assumes that the pixel ***x*** of a particular class can be represented as a sparse linear combination of a dictionary ***D***. Then ***x*** can be sparsely represented as ***x*** = ***Dα*** or approximate ***x*** ≈ ***Dα*** , satisfying


(2) $${\left\|x-D\alpha \right\|}_{2}\leq e$$

where ***α*** ∈*ℝ* ^*M*×1^ contains the representation coefficients for the pixel ***x*** and *e* is the residual.

The sparse coefficient vector $\hat{\alpha }$ can be obtained by solving the following optimization problem:


(3) $$\hat{\alpha }=\text{arg}\underset{\alpha }{\min}{\left\|x-D\alpha \right\|}_{2}\;\;\text{s}.\text{t}.{\left\|\alpha \right\|}_{0}\leq K_{0}$$

where ∥·∥_0_ represents the *l*_0_-norm of the vector ***α*** which counts the number of nonzero entries in the vector and *K*_0_ is the upper bound of the sparse level which is equal to the number of nonzero rows in $\hat{\alpha }$ . The solution with the fewest number of nonzero coefficients is certainly an appealing representation. However, equation (3) is NP-hard (Li et al., 2017), which can be approximately solved by matching pursuit algorithms. Once $\hat{\alpha }$ is obtained, the class of the pixel ***x*** is determined as the one with the minimal reconstruction residual (Fang et al., 2017),


(4) $$\text{class}(\text{x})=\arg\underset{c=1,\cdots ,C}{\min}{\text{r}}^{c}(\text{x})=\arg\underset{c=1,\cdots ,C}{\min}\|\text{x}-\text{D}{\hat{\alpha }}_{c}{\|}_{2}$$

where ${\hat{\alpha }}_{c}$ is the sparse coefficient subset of $\hat{\text{α}}$ belonging to *c*-th class.

### 3.2 Proposed method

#### 3.2.1 Algorithm framework

**Figure 2:**
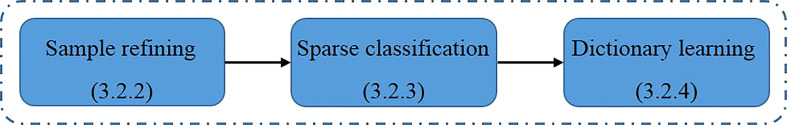
The graphical representation of the overall process of the proposed GSC algorithm.

The proposed method contains three processes: sample refining, dictionary learning, and sparse classification. The graphical representation of the overall process of the proposed GSC algorithm is shown in in **Figure 2**.

#### 3.2.2 Sample refining

The sample refining process is used to remove the unqualified training samples (Gong et al., 2019; Lv et al., 2019). For each class, the strategy is manually selecting a set of qualified training samples first, which are referred to as sample seeds in this study. These seeds are then employed as the baseline to be compared with each training sample. If a training sample has insufficient correlation with all seeds, the sample is considered not to belong to this class, and therefore, it will be removed from the training samples. The detailed process is summarized in **Algorithm 1**. In this algorithm, the correlation between a seed and a training sample is evaluated based on the 2-norm, expressed as $∥{\text{z}}_{j}-{\text{v}}_{c,i}{∥}_{2}^{2}/∥{\text{z}}_{j}{∥}_{2}^{2},$ where ***z*** *_j_*–***v*** *_c,i_* is the residual between the seed ***v*** *_ci_* and the training sample ***z*** *_j_*. The ratio $∥{\text{z}}_{j}-{\text{v}}_{c,i}{∥}_{2}^{2}/∥{\text{z}}_{j}{∥}_{2}^{2}$ is larger than the threshold γ means ***z*** *_j_* has insufficient correlation to ***v*** *_c,i_*. The above evaluation of ***z*** *_j_* is repeated for all seeds. If ***θ*** *_min_ ≥* **γ**, i.e., ***z*** *_j_* has insufficient correlation to all seeds, the training sample ***z*** *_j_* will be removed. The algorithm is executed for the peeling-damaged class and sound class to obtain training samples ***Z***_1_∈*ℝ* ^*n*_*p*_×*η*_1_^ and ***Z***_2_∈*ℝ* ^*n*_*p*_×*η*_2_^ , respectively, where *η*_1_ and *η*_2_ denote the number of training samples of two classes, respectively.

Algorithm 1Sampling refining process of the GSC.
**Input**: Original training samples *
**Z**
*∈*ℝ*
^
*n*
_
*p*
_×*η*
^ , **
*V*
** = [**
*z*
**
_1,_
**
*z*
**
_2_,⋯,**
*z*
**
*
_η_
*], threshold *γ*

**Initialize**:Sample seeds *
**V**
* ∈ *ℝ*
^
*n*
_
*p*
_×*ρ*
^, *
**V**
*=[*
**ν**
*
_1_, *
**ν**
*
_2_,⋯,*
**ν**
*
_
*ρ*
_] ;
**1. For** each training sample **
*z*
**
*
_j_
* **do2.**


$\theta_{min}=\underset{i}{\min}(∥z_{j}-\nu_{c,i}{∥}_{2}^{2}/∥z_{j}{∥}_{2}^{2}),\;i=1,2,\cdots ,\rho$

;
**3. If** *θ_min_
* ≥ γ
**4.** Remove **
*z*
**
*
_j_
* from the training samples;
**Output**: Refined training samples **
*Z*
**

#### 3.2.3 Dictionary learning

Next, the dictionary learning process is executed to obtain the sparse dictionaries of peeling-damaged class and sound class, respectively. The process is summarized as **Algorithm 2**. Given the training samples ***Z***, The first step is to initialize the dictionary ***D*** ∈*ℝ* ^*n*_*p*_×*q*^ , where *q* represents the number of atoms(column) of ***D***. In this study the dictionary is initialized to be the Gaussian random matrix. The training process is an iterative process with the maximum number of iterative cycles of *t_max_*. For each iterative cycle, the sparse coefficients ***s***_j_ is computed based on the *l*_0_-optimization problem, $s_{j}=\arg\underset{s}{\min}∥z_{j}-\text{D}\text{s}{∥}_{2}\;\;\text{s}.\text{t}.∥\text{s}{∥}_{0}\leq k$ , which can be solved by algorithms such as the orthogonal matching pursuit (OMP) (Wang et al., 2012). The step of sparse coefficients computation is called sparse coding. By completing the sparse coding step, the matrix ***S*** = [***s***_1_, ***s***_2_, ⋯, ***s***_ζ_] is obtained. Then, for each atom ***d*** *_i_*, we inspect whether the atom has been used in the sparse coding step. If an atom was not used, it will be removed from the dictionary, shown as lines 7–8 in **Algorithm 2**. The other atoms are then updated by using the strategy of the K-SVD. The **Algorithm 2** is executed for both two classes, respectively, and two dictionaries ***D***_1_∈*ℝ* ^*n*_*p*_×*q*_1_^ and ***D***_2_∈*ℝ* ^*n*_*p*_×*q*_2_^ , where *q*_1_ and *q*_2_ denote the number of atoms of two dictionaries, respectively. It should be pointed out that the background, i.e., the pixels that do not belong to peeling-damaged class or sound class, usually contains various features. Thus, it is difficult to obtain a dictionary that can represent all features accurately. Based on this consideration, the background class is not involved in the dictionary learning process, but the classification among the background class and other two classes can be still realized, which is described as follows.

Algorithm 2 Dictionary learning process of the GSC.
**Input**: Training samples *
**Z**
*∈*ℝ*
^
*n*
_
*p*
_×*ξ*
^ , sparse level *k*, maximum number of iterative cycles *t_max_
*, initial overcomplete level *β*
**Initialize**: *t*=0, *q*=*βn*
_
*p*
_, *
**D**
*=[*
**d**
*
_1_,*
**d**
*
_2_,⋯,*
**d**
*
_
*q*
_], *
**D**
*∈*ℝ*
^
*n*
_
*p*
_×*q*
^ ;
**1. While** *t* ≤ *t_max_
* **do2.** t = t + 1;
**3.** *
**S**
*=[*
**s**
*
_1_,*
**s**
*
_2_,⋯,*
**s**
*
_
*ξ*
_]=*
**O**
*, *
**S**
*∈*ℝ*
^
*q*×*ξ*
^ ;
**4. For** *j* = 1 to ζ **do5.** Solve 


${\text{s}}_{j}=\text{arg}\underset{s}{\text{min}}∥{\text{ z}}_{j}-\text{D}\text{s}{∥}_{2}\;\;\text{s}.\text{t}.∥\text{s}{∥}_{0}\leq k$

;
**6. For** each atom **
*d*
**
*
_i_
* **do7. If *S*
** (*i*,:) contains only zero entries
**8.** Remove **
*d*
**
*
_i_
* from the dictionary;
**9. Else** Update **
*d*
**
*
_i_
* by using the K-SVD strategy;
**10.** Update *q*;
**Output**:Dictionary **
*D*
**

#### 3.2.4 Sparse classification

The obtained dictionaries are used for pixel level classification of HSIs. The algorithm is summarized in **Algorithm 3**. The matrix ***Y*** denotes the classification result of the HSI $\underline{\text{X}}$ , and it is initialized by the zero matrix (all entries of ***Y*** are zero). The classification process is realized by classifying each spatial pixel. Given an arbitrary spatial pixel, the spectral data is extracted from $\underline{\text{X}}$ , expressed as $\text{x}=\underline{\text{X}}\left(i,j,:\right)$ . Then, the sparse recovery problems with respect to ***x*** and two dictionaries ***D***_1_ and ***D***_2_ are solved to obtain the sparse coefficients ***s***_1_ and ***s***_2_, respectively, shown as steps 3 and 5 in **Algorithm 3**. Similar to the sparse coding process in **Algorithm 2**, the sparse recovery problem can be solved by using the OMP algorithm or other *l*_0_-optimization algorithms. The residuals of sparse recovery results are computed, denoted as ***r***_c_ = ***x*** – ***D***_c_ ***s***_c_, c = 1, 2. The 2-norm of residuals are computed, denoted as *e*_1_ and *e*_2_, respectively. Then, the classification of the pixel is determined by the judgement given by steps 7 and 8 of **Algorithm 3**. If the smaller one of *e*_1_ and *e*_2_ is larger than threshold *ε*, it means the spectral data ***x*** cannot be accurately represented by neither ***D***_1_ nor ***D***_2_. Hence, the pixel is considered to belong to neither peeling-damaged class nor sound class, and it is determined to belong to the background class (denoted as value-0 in ***Y***). If the smaller one of *e*_1_ and *e*_2_ is not larger than threshold *ε*, the pixel is determined to belong to the class that satisfies $\arg\underset{\text{c}}{\min}\{e_{\text{c}}\}$ . The steps 2–8 are repeated for each spatial pixel, and finally the classification result ***Y*** ∈*ℝ* ^*n*_*s*_1__×*n*_*s*_2__^ is obtained.

Algorithm 3 Sparse classification process of the GSC.
**Input**: HSI 


$\underline{\text{X}}\in {\mathbb{R}}^{n_{s_{1}}\times n_{s_{2}}\times n_{p}}$
, dictionaries **
*D*
**
_1_, **
*D*
**
_2_ sparse level *k*, threshold *ε*

**Initialize**:


$\text{Y}=\text{O}\in {\mathbb{R}}^{n_{s_{1}}\times n_{s_{2}}}$
;
**1. For** each spatial pixel (*i*, *j*) **do2.** 


$\text{x}=\underline{\text{X}}\left(i,j,:\right)$
;
**3.** Solve 


${\text{s}}_{1}=\arg\underset{s}{\min}∥\text{x}-{\text{D}}_{1}\text{s}{∥}_{2}\;\;\text{s}.\text{t}.∥{\text{s}∥}_{0}\leq k$
;
**4.** Compute **
*r*
**
_1_ = **
*x*
** – **
*D*
**
_1_
**s**
_1_, *e*
_1_ = ∥**
*r*
**
_1_∥_2_;
**5.** Solve


${\text{s}}_{2}=\arg\underset{s}{\min}∥\text{x}-{\text{D}}_{2}\text{s}{∥}_{2}\;\;\text{s}.\text{t}.∥{s∥}_{0}\leq k$
;
**6.** Compute **
*r*
**
_2_ = **
*x*
** – **
*D*
**
_2_
**s**
_2_, *e*
_2_ = ∥**
*r*
**
_2_∥_2_;
**7. If** min (*e*
_1_, *e*
_2_) ≤ *ε*
**8.** 


$\text{Y}\left(i,j\right)=\arg\underset{c}{\min}\{e_{c}\}\;$
;
**Output**:Classification result **
*Y*
**

#### 3.2.5 Evaluation index

The classification result of the HSI was obtained by pixel level classification. Considering that some outliers might reduce the accuracy of the algorithm, the block level calculation method was adopted to process the original classification result and eliminate the influence of outliers in this paper. In the block level method, first of all, every 2×2 pixels in the prediction classification results were divided into small blocks; and then the class with the largest number of pixels in the small block was statistically obtained; finally, all pixels in this small block were divided into this class. The evaluation indexes were the overall classification accuracy and the pixel prediction accuracy. The overall classification accuracy is the percentage of the correctly classified sound fresh corn and damaged fresh corn in the training set and test set. The pixel prediction accuracy of the entire HSI region can be calculated by dividing the number of the correctly predicted pixels in the classification result by the total number of the pixels in the test image. The pixel prediction accuracies of the damaged region can be calculated by dividing the number of correctly predicted pixels in the damaged region by the total number of such pixels in the ground-truth.

## 4 Experimental results and analysis

The experiment results and discussion are introduced in this section. Firstly, the refined training samples are presented, and the characteristics of reflectance spectra of fresh corn are analyzed. Then, the key parameters of the proposed GSC method are determined through experiments. Finally, the classification results of fresh corn HSIs are described. All experiments were carried out using the Matlab 2021a software.

### 4.1 Refined samples and spectral characteristic

**Figure 3:**
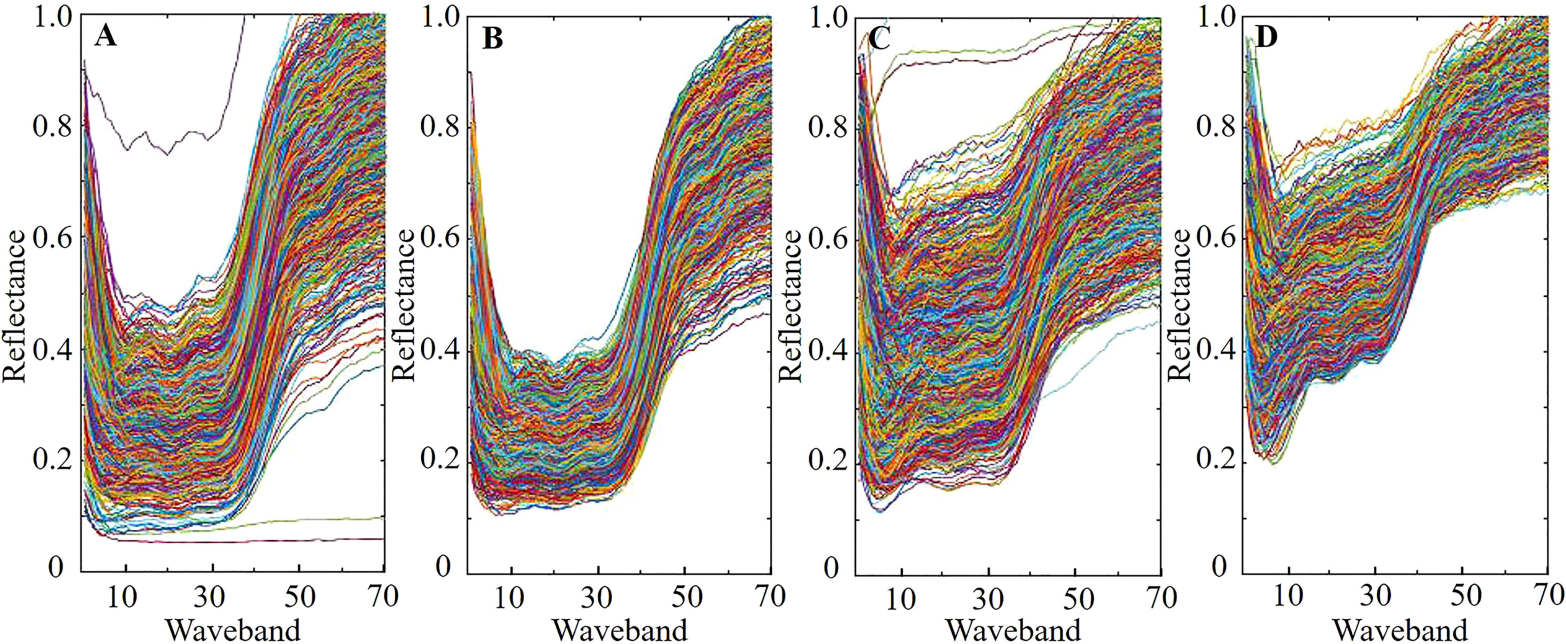
Original training samples and refined training samples of the sound class and peeling-damaged class of fresh corn under the first 70 wavebands: **(A)** original training samples of sound class; **(B)** refined training samples of sound class; **(C)** original training samples of peeling-damaged class; **(D)** refined training samples of peeling-damaged class.

There were 146893 and 190242 training samples for sound class and peeling-damaged class of fresh corn, respectively. After the sample refining process, 39085 and 116427 impure training samples were removed, and then 107808 and 73815 refined training samples were left in sound class and peeling-damaged class, respectively. The same refined training samples were used in the GSC, K-SVD, SVM, and BP classification methods. **Figure 3** shows the original training samples and the refined training samples of the sound class and peeling-damaged class of fresh corn. As shown in **Figure 3**, some obviously unqualified training samples in the sound and peeling-damaged classes had been removed after sample refining. In addition, it could be seen that the spectral reflection intensity decreased first and then increased. The relative intensity of the peeling-damaged fresh corn was lower than that of the sound one, and it was because the damaged pigment and collapsed tissue of fresh corn could cause a reduced light reflection (Gao et al., 2019).

### 4.2 Experimental parameter selection

**Figure 4:**
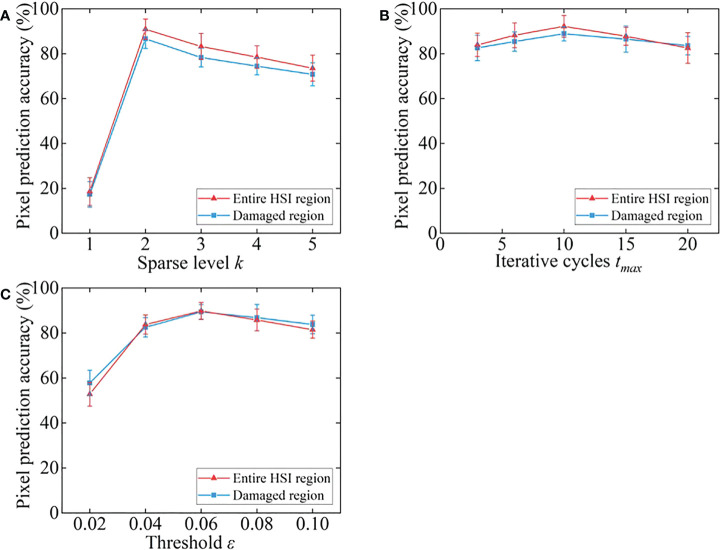
Average pixel prediction accuracies under different parameter levels. **(A)** sparsity level *k*; **(B)** iterative cycles *t_max_*; **(C)** threshold *ϵ*.

**Figure 5:**
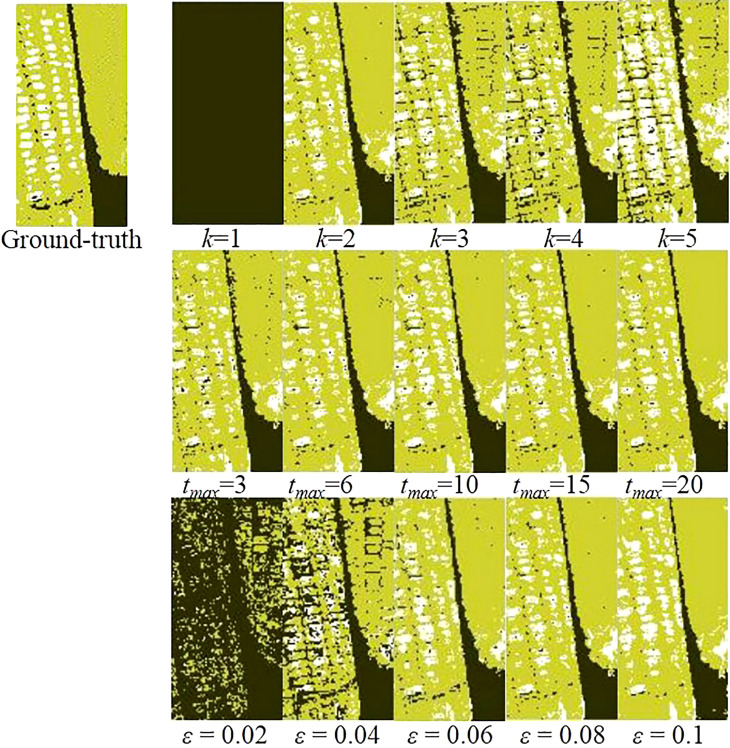
Classification maps under different parameter levels.

In order to obtain better classification performance, some key parameters of the proposed GSC methods needed to be determined, such as the sparse level *k*, maximum number of iterative cycles *t_max_*, and threshold *ε*. The optimal parameters were determined by experiments, *k* ranged in {1, 2, 3, 4, 5}, *t_max_* ranged in {3, 6, 10, 15, 20}, and *ε* ranged in {0.02, 0.04, 0.06, 0.08, 0.10}. If the parameter values were selected appropriately, the pixel prediction accuracies were high. Therefore, different parameter values were tested repeatedly on 12 HSIs of peeling-damaged fresh corn selected from the test set to determine the appropriate values of *k*, *t_max_*, and *ε*. The following parameter combinations were selected during the test: the sparsity level was 2, maximum number of iterative cycles was 15, and threshold was 0.08. Two parameters were fixed in each group of tests to explore the appropriate parameter values for maximizing the pixel prediction accuracies of the GSC method. The average pixel prediction accuracies under different parameters are displayed in **Figure 4**. Aiming for the highest average pixel prediction accuracies, the optimal parameter values of the proposed GSC method were finally set as *k*=2, *t_max_* =10, and *ε* =0.06 based on the experimental results shown in **Figure 4**. Taking one HSI as an example, the classification maps under different parameters are shown in **Figure 5**. The yellow pixels in classification maps represented the sound corn, the white pixels represented the peeling-damaged corn, and the black pixels represented the background.

### 4.3 Classification results analysis

In this subsection, the GSC method and other three commonly used K-SVD, SVM, and BP methods were used to conduct the overall classification of fresh corn HSIs in the training set and test set, and finally determined that the fresh corn belonged to sound or damaged ear. Besides, all test fresh corn HSIs in the test set were precisely classified, and each pixel in the image was classified at the pixel level. Finally, the pixel was ascertained to belong to sound kernel, damaged kernel, or background classes. The overall classification accuracy and average pixel prediction accuracy of the four methods were obtained and compared.

#### 4.3.1 Results on overall classification accuracy

The overall classification accuracy results of the fresh corn HSIs in the training set and test set using four classification methods are listed in **Table 2**. It can be seen that the proposed GSC method performed best in distinguishing sound and damaged fresh corn ears. For the training set, the identification accuracy of sound and damaged fresh corn by GSC method was 98.33%, with 2 damaged fresh corns misjudged as sound fresh corns. For the test set of the GSC method, the identification accuracy of sound and damaged fresh corn by was 95.00%, with 1 sound fresh corn misjudged as damaged fresh corn and 2 damaged fresh corns misjudged as sound fresh corns. Dysplastic corn kernels on fresh corn were often identified as damaged class, resulting in the sound fresh corn might be misjudged as the damaged fresh corn. In addition, the damaged fresh corn was misjudged as the sound fresh corn because the chemical and physical information on the surface tissue did not change significantly when the seed coat of fresh corn was slightly damaged. At this time, the spectral curves of the damaged fresh corn were similar to that of the normal fresh corn. Qiao et al., (2021) drew the same conclusion in the nondestructive detection of decayed blueberry.

**Table 2:** Overall classification accuracies of HSIs in the training/test set using four methods under the selected optimal first 70 wavebands.

Classification method	Training set (120)			Test set (60)		
	Sound (60)	Damaged (60)	Accuracy (%)	Sound (30)	Damaged (30)	Accuracy (%)
GSC	60	58	**98.33**	29	28	**95.00**
K-SVD	54	49	85.83	26	23	81.67
SVM	53	52	87.50	24	26	83.33
BP	54	57	92.50	26	28	90.00

The bold numbers represent the maximum values of the overall classification accuracy of the four classification methods.

#### 4.3.2 Results on average pixel prediction accuracy

**Figure 6:**
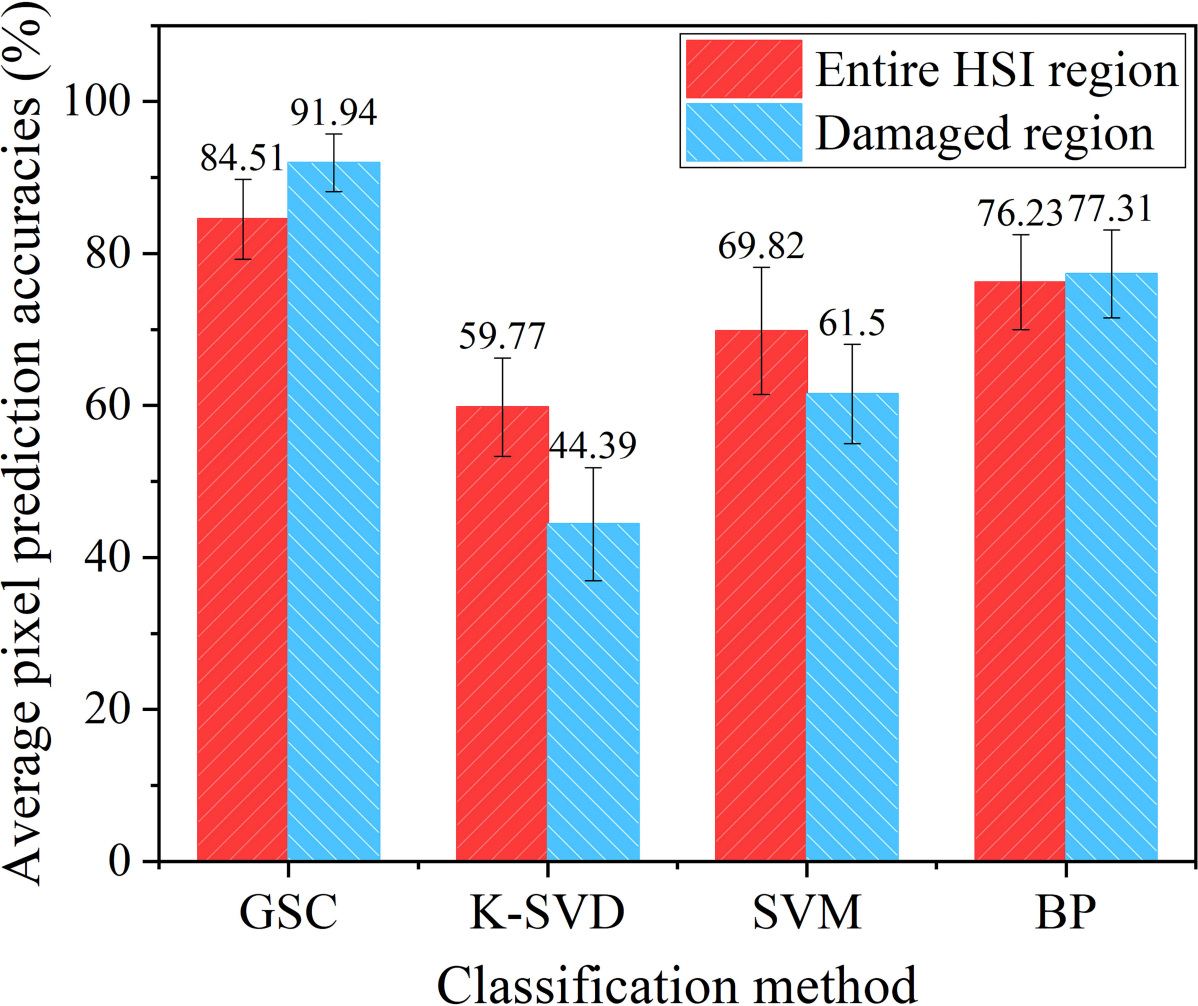
Average pixel prediction accuracies of the HSIs in the test set using four methods based on the given ground-truths.

Four classification methods were applied to classify the test fresh corn HSIs in the test set for precise pixel classification, and the average pixel prediction accuracies of 60 test images are shown in **Figure 6**. For the pixel classification results of the entire HSI region, the proposed GSC method had the highest average pixel prediction accuracy of 84.51%, followed by the BP neural network method which reached 76.23%. The average pixel prediction accuracy of the GSC method was 41.39%, 21.04%, and 10.86% higher than that of K-SVD, SVM, and BP methods, respectively. For the pixel classification results of the damaged region, the proposed GSC method had the highest average pixel prediction accuracy of 91.94%, followed by the BP, K-SVD and SVM methods with 77.31%, 61.50%, and 44.39%, respectively. The average pixel prediction accuracy of the GSC method was at least 18.92% higher than that of other methods. The average pixel prediction accuracy of the entire HSI region of four methods including the GSC method did not reach above 85%, but this did not affect the practical application of the GSC method. This is because the accurate identification of the damaged region of fresh corn in the practical application process is the key to realize the automatic detection and grading of peeled fresh corns. However, the average pixel prediction accuracy of the GSC method for the damaged region was higher than 90%, which satisfied the practical application.

#### 4.3.3 Analysis of classification results on typical scenes

**Figure 7:**
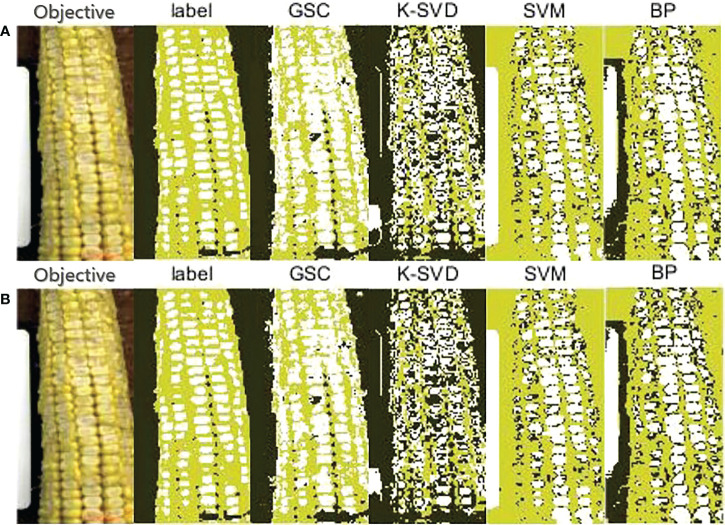
Classification results on a single seriously damaged fresh corn in Scene 1. **(A)** Pixel-level; **(B)** 2×2 block-level.

**Figure 8:**
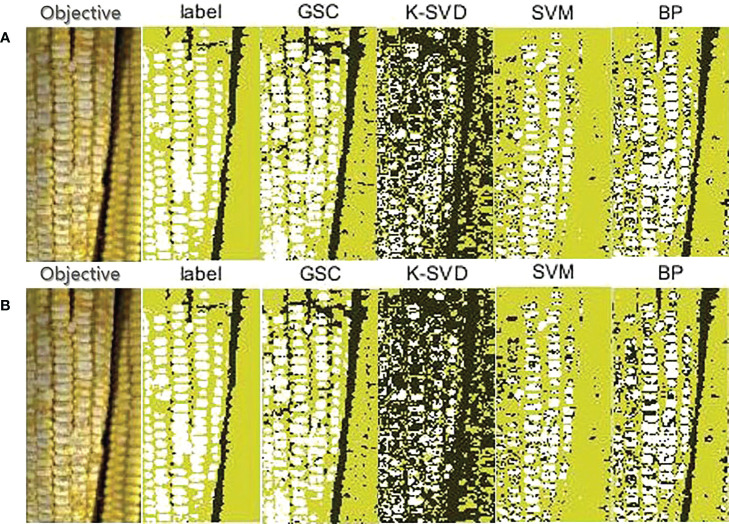
Classification results on one sound and one seriously damaged fresh corns in Scene 2. **(A)** Pixel-level; **(B)** 2×2 block-level.

**Figure 9:**
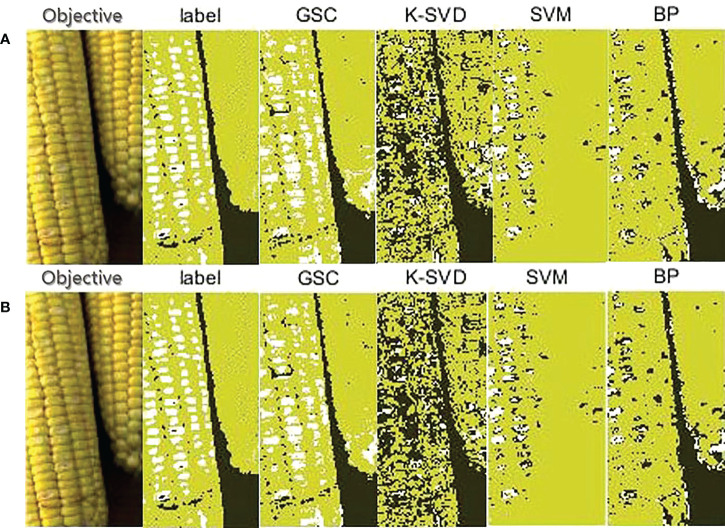
Classification results on one sound fresh corn and one damaged fresh corn with slight abrasion of seed coats in Scene 3. **(A)** Pixel-level; **(B)** 2×2 block-level.

Three typical fresh corn HSIs were selected from the test set for detailed study, and denoted as Scene 1, Scene 2, and Scene 3, respectively. The classification results by using four methods of the selected three scenes are presented in **Figures 7**–**9**. Each method had two classification result images. One was the original classification result image based on pixel-level classification, the other was the classification result image processed by the 2×2 block-level method. In the following analysis, the pixel prediction accuracy of each method was considered to be the larger value of the two classification results. The RGB images corresponding to fresh corn HSIs (‘Objective’) and the corresponding ground-truths with manual labeling (‘label’) were given as the reference images of classification results in **Figures 7**–**9**.

(1) Classification results on Scene 1

There was a seriously damaged fresh corn ear in Scene 1, in which the seed coats of most kernels had disappeared and the liquid endosperm was exposed. **Table 3** shows the pixel prediction accuracy of Scene 1. Among the four methods, the GSC method had the highest pixel prediction accuracy of 78.77%, while the SVM method had the lowest pixel prediction accuracy of 54.41%. The prediction accuracy values of Scene 1 using K-SVD, SVM, and BP methods were all lower than the average values given in **Figure 6**. The possible reason is that the pure Teflon whiteboard as background class was wrongly classified as damaged kernel class by the three methods. As shown in **Figure 7**, the GSC and K-SVD methods could identify the complete contour of the objective fresh corn, while the SVM and BP methods incorrectly classified most of the background pixels into sound kernel class. The pixel prediction accuracy of the GSC method for the damaged regions of fresh corn in Scene 1 was 92.89%. The GSC method could recognize almost all damaged kernels, while the SVM and BP methods could only recognize a part of damaged kernels. However, the K-SVD method misclassified most of damaged kernels into background class.

**Table 3:** Pixel prediction accuracies of the four methods based on the given ground-truth of Scene 1.

Prediction accuracy (%)	Classification method			
	GSC	K-SVD	SVM	BP
Pixel-level	78.20	57.85	54.41	59.38
Block-level	**78.77**	56.55	52.19	61.14

The bold numbers represent the maximum values of the pixel prediction accuracy of the four classification methods.

(2) Classification results on Scene 2

There were two fresh corn ears in Scene 2. One was a sound ear and the other was a seriously damaged ear. **Table 4** shows the pixel prediction accuracy of Scene 2. Among the four methods, the GSC method had the highest pixel prediction accuracy of 85.15%, while the K-SVD method had the lowest pixel prediction accuracy of 45.66%. As shown in **Figure 8**, first, the GSC method could detect the complete contours of the two fresh corns and background, this indicated that the GSC method had the potential of detecting multiple fresh corns; second, it could identify almost all the damaged kernels in the damaged fresh corn; and third, it could completely recognize all the sound kernels in the sound fresh corn, in addition to identifying the gaps between kernels as background. The BP method could also identify the regions of fresh corns and background, but it could only identify a part of damaged kernels. The SVM method could not detect the edges of fresh corns, and they wrongly classified the background between two fresh corns into sound kernel class. The K-SVD method could identify the complete region of background, but it misclassified almost all damaged kernels into background class.

**Table 4:** Pixel prediction accuracies of the four methods based on the given ground-truth of Scene 2.

Prediction accuracy (%)	Classification method			
	GSC	K-SVD	SVM	BP
Pixel-level	84.83	45.66	73.17	79. 39
Block-level	**85.15**	42.14	74.99	77.10

The bold numbers represent the maximum values of the pixel prediction accuracy of the four classification methods.

(3) Classification results on Scene 3

There were two fresh corn ears in Scene 3. One was a sound ear and the other was a damaged ear with slight abrasion of seed coats. **Table 5** shows the pixel prediction accuracy of Scene 3. Among the four methods, the GSC method had the highest pixel prediction accuracy of 87.68%, while the K-SVD method had the lowest pixel prediction accuracy of 65.85%. As shown in **Figure 9**, the GSC methods could accurately and completely detect fresh corns and background with clear edges, but it wrongly identified dysplastic corn kernels on the sound ear as damaged class. The K-SVD and BP methods could also effectively distinguish the fresh corn region and background region, but there were more pixels misclassified at the edge of fresh corn. Similar to the situation in Scene 2, the K-SVD method still misclassified the damaged kernels into background class, and the SVM method still misclassified the background between two fresh corns into sound kernel class. The SVM and BP methods could only identify a few damaged kernels.

**Table 5:** Pixel prediction accuracies of the four methods based on the given ground-truth of Scene 3.

Prediction accuracy (%)	Classification method			
	GSC	K-SVD	SVM	BP
Pixel-level	86.87	65.85	73.91	81.42
Block-level	**87.68**	62.07	73.64	82.14

The bold numbers represent the maximum values of the pixel prediction accuracy of the four classification methods.

### 4.4 Discussion

#### 4.4.1 Discussion on using HSI detection instead of traditional RGB image

It should be noted that the HSI was chose for damage identification of fresh corn instead of the traditional RGB images in this paper, mainly for the following reasons. Traditional RGB image classification method mainly relies on two points. (1) Differences in colors. However, there are many varieties of fresh corn, and the colors of seed coats are diverse, such as yellow, white, purple, and even multiple colors on an ear. Moreover, the color of white seed coat is the same as that of inner endosperm, so the classification accuracy based on the color differences is not high. Therefore, it is not feasible to accurately identify fresh corn peeling-damage by color differences. (2) Differences in spatial characteristics. However, the shape of the damage regions caused by mechanical peeling is random, which may be triangular, circular, or irregular. Therefore, there are no fixed spatial geometric characteristics, and it is not feasible to accurately identify fresh corn peeling-damage using spatial geometric characteristics.

#### 4.4.2 Discussion on dimensionality reduction method

The dimensionality reduction method is to reduce the computation amount and improve the classification accuracy. For example, principal component analysis (PCA), a commonly used feature reduction method, takes training data and test data as a whole to extract the principal components. The results obtained in this case are good for both the training data and the test data. Therefore, the dimensionality reduction data are used for training to obtain the principal components, which are used to classify the test data and get better classification effect. For the problem addressed in this study, the test data needed to deal with cannot be obtained in advance while executing the training process, and therefore, the PCA results obtained by using training data may be not suitable for test data.

#### 4.4.3 Discussion on comparison with existing methods

It could be seen from the experimental results that the developed GSC method had an absolute advantage in hyperspectral detection of fresh corn damage when background contents were containing multiple features. This can be explained at a theoretical level. As shown in **Figure 7**, the GSC method could correctly classify the pure Teflon whiteboard, large gaps between kernels, and marks on fresh corns made by a marking pen into the background class, while the other three methods had poor extraction effect for the background. This is because the GSC method determines background pixels by the threshold algorithm with respect to the energy of sparse representation residual during the sparse classification process. Even if the background contents are not fixed in different detecting situations, the classification effect will not be affected. It is worth mentioning that it is always a challenge to detect the slight abrasion of seed coats. By comparing the classification results in **Figures 8**, **9**, it can be seen that the recognition effect of the fresh corn with slight abrasion of seed coats is obviously inferior to that of the seriously damaged fresh corn, and there was still a gap between the detection result of the abrasion region and the ground-truth. However, compared with other classification methods, the GSC method had the best recognition effect on the abrasion region with the pixel prediction accuracy of 81.13%, while the other three methods almost fail in the abrasion detection. This proves the absolute superiority of our proposed method. One possibility is that the reflectance spectra of slightly bruised seed coats are closer to sound seed coats, thus increasing the difficulty of classification. Another possibility is that it is difficult to recognize slightly bruised kernels with naked eyes, resulting that the ground-truths (‘label’) of the RGB images by manually labeling may be not completely accurate.

#### 4.4.4 Discussion on the practical applications

**Figure 10:**
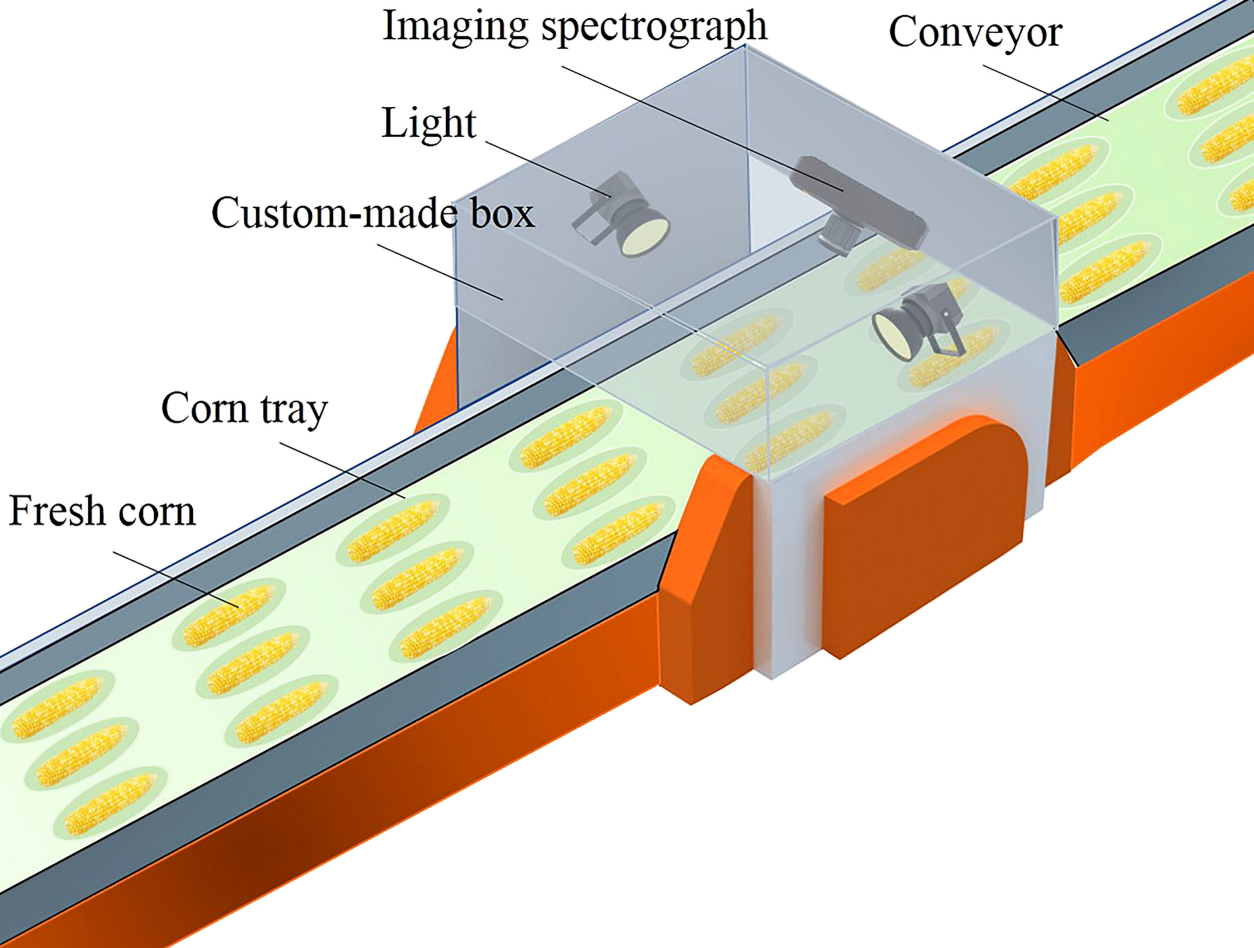
Schematic diagram of the practical applications.

During fresh corn processing, online detection of mechanical damage of fresh corn based on the GSC algorithm can be realized by using the imaging spectrograph. The peeled fresh corns are transferred to the sorting equipment by a conveyor covered with corn trays, and then vacuum packed. In order to improve the quality of fresh corn, damage detection is carried out before sorting. The lighting system and imaging spectrograph are mounted in a custom-made box above the conveyor. The speed of the conveyor is adjusted according to the spatial resolution and the integration time of the imaging spectrograph. The hyperspectral data on the front of the fresh corn are collected when the fresh corn is transported to the bottom of the imaging spectrograph. Then, the corn trays rotate and drive the fresh corns to rotate 180° to collect the hyperspectral data on the opposite side of the fresh corn. The damage detection of the whole fresh corn ear can be realized. Schematic diagram of the practical applications is shown in **Figure 10**.

## 5 Conclusions

In this paper, the feasibility of using hyperspectral imaging technique to detect the fresh corn peeling damage was studied. The GSC method was proposed to classify the pixels of sound kernel, peeling-damaged kernel, and background. For this purpose, each process of the GSC method was introduced, including sample refining, dictionary learning, and sparse classification. The classification results of fresh corn HSIs with serious damage and slight abrasion of seed coats were also presented. Although complete extraction of damage regions in fresh corn ear with slight abrasion of seed coats was still a challenge, the experimental results demonstrated that the GSC method had the highest accuracy regardless of the damage degrees of test images. Experimental results verified the feasibility of the GSC method. The overall classification accuracy of the training set was 98.33%, and that of the test set was 95.00%. It also had the highest average pixel prediction accuracy of 84.51% for the entire HSI regions and 91.94% for the damaged regions, which were significantly higher than compared methods, including the K-SVD, SVM, and BP methods. The peeling-damaged regions of fresh corn could be directly observed by the classification results based on pixel-level classification. This study made up for the gap in the detection method of fresh corn peeling damage. The datasets used for hyperspectral fresh corn damage detection study was built. In general, the results confirmed the feasibility of hyperspectral imaging technique in detecting the fresh corn peeling-damage in laboratory environment.

## Data availability statement

The original contributions presented in the study are included in the article/supplementary material, further inquiries can be directed to the corresponding author/s.

## Author contributions

ZL: Conceptualization, Methodology, Software, Writing-original draft preparation. JF: Resources, Writing-review and editing, Project administration, Funding acquisition. QF: Validation, Investigation. ZC: Supervision. XL: Supervision. All authors contributed to the article and approved the submitted version.

## Funding

This work was supported by the National Natural Science Foundation of China (No. 52105257).

## Acknowledgments

The authors are grateful for the fresh corn provided by the Fumin Food Processing Plant.

## Conflict of interest

The authors declare that the research was conducted in the absence of any commercial or financial relationships that could be construed as a potential conflict of interest.

## Publisher’s note

All claims expressed in this article are solely those of the authors and do not necessarily represent those of their affiliated organizations, or those of the publisher, the editors and the reviewers. Any product that may be evaluated in this article, or claim that may be made by its manufacturer, is not guaranteed or endorsed by the publisher.
